# Epitaxial Growth of Flower-Like MoS_2_ on One-Dimensional Nickel Titanate Nanofibers: A “Sweet Spot” for Efficient Photoreduction of Carbon Dioxide

**DOI:** 10.3389/fchem.2022.837915

**Published:** 2022-01-27

**Authors:** Haritham Khan, Suhee Kang, Hazina Charles, Caroline Sunyong Lee

**Affiliations:** ^1^ Department of Materials and Chemical Engineering, Hanyang University, Ansan, South Korea; ^2^ POSCO Chemical, Sandan-gil, Jeonui-myeon, Pohang, South Korea

**Keywords:** artificial photosynthesis, CO_2_ reduction, hydrophobic nature, Mos_2_, NiTiO_3_, electrospining

## Abstract

Herein, a full spectrum-induced hybrid structure consisting of one-dimensional nickel titanate (NiTiO_3_) nanofibers (NFs) decorated by petal-like molybdenum disulfide (MoS_2_) particles was designed through a facile hydrothermal method. The key parameters for tailoring the morphology and chemical, surface, and interfacial properties of the heterostructure were identified for efficient and selective conversion of CO_2_ into valuable chemicals. Introducing MoS_2_ layers onto NiTiO_3_ NFs provided superior CO_2_ conversion with significantly higher yields. The optimized hybrid structure produced CO and CH_4_ yields of 130 and 55 μmol g^−1^ h^−1^, respectively, which are 3.8- and 3.6-times higher than those from pristine NiTiO_3_ nanofibers (34 and 15 μmol g^−1^ h^−1^, respectively) and 3.6- and 5.5-times higher than those from pristine MoS_2_ (37 and 10 μmol g^−1^ h^−1^, respectively). This improved performance was attributed to efficient absorption of a wider spectrum of light and efficient transfer of electrons across the heterojunction. Effective charge separation and reduced charge carrier recombination were confirmed by photoluminescence and impedance measurements. The performance may also be partly due to enhanced hydrophobicity of the hierarchical surfaces due to MoS_2_ growth. This strategy contributes to the rational design of perovskite-based photocatalysts for CO_2_ reduction.

## Introduction

Global warming due to excessive emission of anthropogenic carbon dioxide has become an increasingly serious environmental concern. It is therefore imperative to develop strategies to mitigate CO_2_ emissions. Exhaustive research has examined sustainable technologies for CO_2_ reduction (Thompson et al., 2020). Photocatalytic CO_2_ reduction is of particular interest, to produce chemical fuels via solar energy conversion, but the activity, stability, and selectivity of the products are strongly dependent on the efficiencies of light-harvesting, charge migration, and surface reactions (Li et al., 2019b).

Nickel titanium trioxide (nickel titanate; NiTiO_3_), a member of the Ti-based perovskite oxide group, has recently received attention due to its photocatalytic activity in visible light (2.1–2.9 eV) (He et al., 2016; Pham and Shin, 2020). NiTiO_3_, with its advantage of octahedrally coordinated Ni and Ti, has a narrow bandgap contrary to that of traditional ultraviolet (UV)-active photocatalysts. Zeng et al. (Zeng et al., 2018) reported nontoxic and low-cost perovskites having more suitable energy bands for CO_2_ reduction and greater stability against photocorrosion. However, recombination of charge carriers occurred when they were used as individual photocatalysts. Effect of heterostructure formation in the semiconductor-based photocatalysts has gained much interest. Semiconductor-based photo-catalysts showed dramatic reduction in the recombination rates with heterostructure formation which are widely applied in antibiotic removal (Li et al., 2021; 2022a), pharmaceutical wastewater treatment (Li et al., 2020b), and toxicity analysis applications (Li et al., 2022b; Wang et al., 2022). Therefore, a composite photocatalyst was required to effectively reduce recombination rates.

Two-dimensional (2D) transition-metal dichalcogenides (TMDs) are another emerging group of materials that show promise because of their unique nanoflower morphology, consisting of layered structures with thin open edges (Chen et al., 2017; Zhang et al., 2020; Gan et al., 2021). TMDs have improved light absorption and charge separation, and hence various catalytic properties. Unlike H_2_ evolution, water treatment, and water splitting, research on CO_2_ reduction performance is still in its infancy. Molybdenum disulfide (MoS_2_; ∼1.3 eV) is the most frequently used TMD having a graphite-like 2D structure. Due to its facile synthesis and cost-effectiveness, MoS_2_ is regarded as an ideal substitute for noble metals in the context of photocatalytic H_2_ evolution. MoS_2_ has three polytypic structures, with the hexagonal 2H and octahedral 1T phases being the most common. The metastable 1T phase, which is active on basal and edge planes, unlike the 2H phase, has interesting chemical and physical properties but its synthesis is challenging (Asadi et al., 2014; Li et al., 2018; Li et al., 2020a; Thomas et al., 2021).

The rational design and preparation of dissimilar dimensional materials (e.g.,1D/2D) has therefore been extensively investigated for use as heterogeneous photocatalysts (Peng et al., 2017; Su et al., 2018; Xu et al., 2018; Li et al., 2020a; Qu et al., 2020). The 1D materials possess distinct advantages in terms of efficient electron transport and optical excitation, but also have the disadvantage of low surface area. Meanwhile, 2D materials exhibit large surface areas but tend to agglomerate. Interfacial engineering is a promising dimensionality-dependent technique for sustainable energy applications. A plethora of photocatalytic studies on the heterostructures of NiTiO_3_/gC_3_N_4_, NiTiO_3_/TiO_2_, Fe_2_O_3_/NiTiO_3_, mono/multilayer MoS_2_, MoS_2_ nanoflowers, 1D/2D TiO_2_/MoS_2_, MoS_2_/graphene, Bi_2_S_3_/MoS_2_, Au-MoS_2_, NiTiO_3_/MoS_2_, phosphated 2D/3D MoS_2_, CdS/MoS_2_, Cu/MoS_2_, and co-doped MoS_2_ nanoparticles have been reported (Chang et al., 2014; Parzinger et al., 2017; Du et al., 2018; Qin et al., 2018; Li et al., 2019c; Lee et al., 2019; Lu et al., 2020; Guo et al., 2021; Khan et al., 2021; Liu et al., 2021) but to the best of our knowledge, no study on CO_2_ reduction via the 1D/2D NiTiO_3_/MoS_2_ structure has been reported.

Herein, a highly synergized NiTiO_3_/MoS_2_ (1D/2D) heterostructure was synthesized using a two-step process. NiTiO_3_ nanofibers (NFs) were firstly synthesized via electrospinning which were later combined with 2D flower-like MoS_2_ via a hydrothermal process. The morphologies and optical properties of the as-synthesized photocatalysts were characterized using various techniques. The selective growth of highly reactive 1T, along with honeycomb-like 2H phases, was confirmed by X-ray photoelectron spectroscopy (XPS) and transmission electron microscopy (TEM). The hybrid NiTiO_3_/MoS_2_ exhibited a redshift to the visible light region, with enhanced absorption. At the optimum loading of MoS_2_, NiTiO_3_/MoS_2_ exhibited the greatest CO_2_ reduction, producing CO and CH_4_ gases at 130 and 55 μmol g^−1^ h^−1^, respectively, which are 3.8- and 3.6-times higher amounts than those (34 and 15 μmol g^−1^ h^−1^, respectively) of pristine NiTiO_3_ NFs, achieving an overall CO_2_ selectivity of 83%. This work could contribute to the development of efficient and stable photocatalytic materials for water splitting, H_2_ evolution, and other photocatalytic activities.

## Materials and Methods

### Materials

All materials were of analytical grade and were used without further purification. Nickel (II) acetate tetrahydrate (98%), titanium (IV) butoxide (97%), sodium molybdate dihydrate (≥99.5%), ethanol (EtOH), and acetylacetone were purchased from Sigma–Aldrich (St. Louis, MO, United States). Polyvinylpyrrolidone K90 (MW = 360,000) and thiourea (H_2_NCSNH_2_), obtained from Wako Pure Chemical Industries, Ltd (Osaka, Japan), were used in the synthesis of the NiTiO_3_ NFs. Sodium sulfate (Na_2_SO_4_) and triethanolamine (TEOA) from Samchun Pure Chemical Co., Ltd (Pyeongtaek-si, Korea) were used in the measurement of electrochemical and photocatalytic properties. Conductive fluorine-doped tin oxide (FTO; 15 mΩ) glass with dimensions of 2 × 6 cm^2^, purchased from Korea Fine Chemical Co., Ltd., was used as a substrate film.

### Preparation of NiTiO_3_ Nanofibers

NiTiO_3_ NFs were made via electrospinning. Briefly, titanium butoxide (2 g) was stirred in EtOH (5 ml) until it was well-mixed. Then, the required amount of nickel (II) acetate tetrahydrate was added and the solution was continuously stirred for 3 h at room temperature. Subsequently, PVP (0.6 g) was added to the solution, which was stirred continuously for another 8 h to obtain a viscous solution. Finally, acetylacetone (0.3 ml) was added and the solution was stirred for at least 2 h until uniformly light green in color. The viscosity of the solution measured using a DV2TLVCJ0 viscometer (AMETEK Brookfield, Chandler, AZ, United States) was 195 ± 5 cP. This solution was taken-up in a 12-ml nonpyrogenic plastic syringe and connected to a 25-gauge (0.26 mm) stainless-steel needle for electrospinning. The syringe was then mounted vertically and attached to a pump that was connected to a high-voltage power supply. The distance from the needle to the collection plate was fixed at 110 mm and the flow rate was maintained at 10 μl/min. Electrospinning was performed using an electrospinning machine (Model ESR100D; NanoNC, Seoul, Korea) and maintained at 10 kV for the synthesis of NiTiO_3_ NFs. The NFs were collected every 2 h and kept in an oven at 60°C, to remove residual solvent before heat treatment in a box furnace under an air atmosphere of 600°C (4 h, 10°C/min). These NFs were characterized without further treatment.

### Preparation of NiTiO_3_/MoS_2_ Structures

**SCHEME 1:**
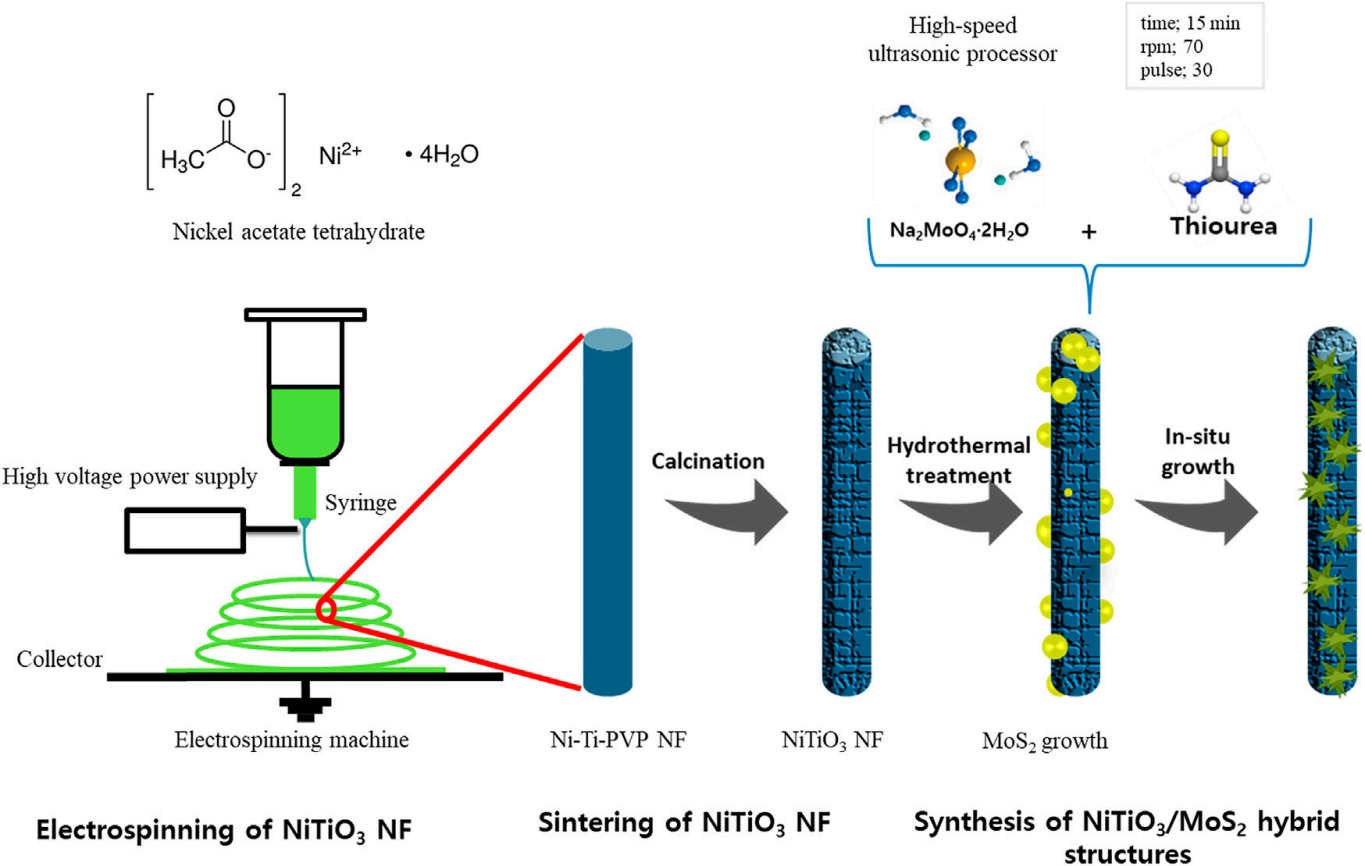
Illustration of the complete fabrication processes of NiTiO_3_ NFs and NiTiO_3_/MoS_2_ hybrid structures.

NiTiO_3_/MoS_2_ with different loading amounts of MoS_2_ precursors was synthesized by a hydrothermal process. The required amounts of sodium molybdate dihydrate and thiourea were dissolved in 60 ml of deionized (DI) water under stirring for 1 h. Then, 100 mg of NiTiO_3_ NFs were added and mixed by a high-speed ultrasonic processor (VCX-130; Young Jin Corporation, Korea) for 15 min (70 rpm, 30-s pulse) to ensure uniform and complete mixing. The solution was then transferred to a 100-ml Teflon-lined autoclave to grow MoS_2_ nanosheets over the NiTiO_3_ NFs via a hydrothermal method. This mixture was maintained at 200°C for 24 h in a box furnace under an air atmosphere. Finally, the NiTiO_3_/MoS_2_ sample was collected and washed three times with DI water and EtOH to remove organic impurities. The obtained black-colored NiTiO_3_/MoS_2_ samples were dried overnight in a vacuum oven at 60°C and then characterized. Scheme 1 illustrates the complete synthesis process. The amount of MoS_2_ precursor was varied and samples were classified as NMS-01, NMS-02, NMS-03, or NMS-04 (Supplementary Table S1). For comparison, pristine MoS_2_ was synthesized under the same process conditions without adding NiTiO_3_ NFs.

### Photoelectrochemical Performance

A three-electrode quartz cell with a potentiostat (VersaSTAT 4; Princeton Applied Research, Princeton, NJ, United States) was used to measure the photoelectrochemical performances of the photocatalysts. Electrolyte (0.5 M Na_2_SO_4_) was used during this process. Each photocatalyst (200 mg) was dissolved in 1.5 ml of EtOH and coated on the 2 × 4 cm^2^ area of the FTO film (2 × 6 cm^2^) via spin-coating at 2,500 rpm for 60 s. The as-prepared films were sintered at 150°C for 1 h to remove residual EtOH. Each coated FTO film contained ∼1.5 mg of the photocatalyst. The FTO film, Pt wire, and Ag/AgCl were used as the working, counter, and reference electrodes, respectively. The films were characterized using electrochemical impedance spectroscopy (EIS) under ultraviolet-visible (UV-vis) light irradiation at frequencies ranging from 10^5^ to 0.1 Hz, at an AC amplitude of 10 mV. The photocurrent density was measured while 1 V bias potential was applied via the reference electrode. All photoelectrochemical analyses were conducted using a 300-W Xe lamp (66984; Newport, Irvine, CA, United States) under UV-vis light irradiation.

### Photocatalytic Activity

The photocatalytic performance of all samples was measured according to the photoreduction of CO_2_ under UV-vis light irradiation. The experiments were carried out in a homemade chemical-resistant quartz-windowed stainless-steel reactor cell (260 ml) equipped with a 300-W Xe lamp as the light source. In a typical procedure, the required amount of sample was coated on the FTO film and used as a photocatalyst to react with CO_2_ and water (125 ml) inside the reactor. Triethanolamine (10 vol%) was used as a hole scavenger. Before photocatalytic experiments, the reactor cell was purged with CO_2_ gas (99.99% purity; 2 bars for 2 h) to remove air and other gases. Evolved gases were collected every hour and separated by a fused silica capillary column equipped with a pulsed discharge detector (6500 GC; YL Instruments, Gyeonggi-do, Republic of Korea). Helium continuously flowed as carrier gas. A 300-W Xe lamp was used as a simulated sunlight source and the focused light intensity (10 mW cm^−2^) was measured using an 843-R USB power meter (MKS; Newport).

### Physicochemical Characterization

The microstructures of pristine NiTiO_3_ NFs, MoS_2_, and NiTiO_3_/MoS_2_ (NMS-X, where X = 1–4) were measured by field-emission scanning electron microscopy (FE-SEM; S4800; Hitachi, Japan) at 15 kV. To further investigate the structure and interaction between NiTiO_3_ NFs and MoS_2_, scanning TEM (STEM; JEM2100F; JEOL, Tokyo, Japan) analysis was performed at 200 kV. X-ray diffraction (XRD; D/Max-2500/PC; Rigaku, Tokyo, Japan) was carried out using a Bruker Advanced X-ray instrument with Cu Kα radiation at a wavelength of 1.5418 Å, to analyze the crystalline phases of the samples. A Brunauer–Emmett–Teller (BET) N_2_ adsorption/desorption analyzer (TriStar II 3020; Micromeritics, Norcross, GA, United States) was used to measure the specific surface area and pore size distribution of the catalysts. To study the chemical properties of the photocatalysts, XPS (Thermo Fisher Scientific, Waltham, MA, United States) with an Al Kα source was used. Optical properties were measured by UV-vis spectroscopy (V750; JASCO, Tokyo, Japan). Fourier transform infrared spectroscopy (FTIR, iS10; Thermo Fisher Scientific) was used to confirm the presence of specific surface groups (NH_2_, OH) in NiTiO_3_ NF and NMS-02. A photoluminescence spectrophotometer (LabRAM HR-800; Horiba, Piscataway, NJ, United States) was used to study the recombination rates of the charge carriers. Contact angles (CAs) were measured at room temperature (22–25°C) and 20–30% RH using a static CA analyzer (Phoenix 300; SEO, Suwon, Republic of Korea). For the analysis, 3.4 µl of DI water was used.

## Results and Discussion

### Field-Emission Scanning Electron Microscopy

**FIGURE 1:**
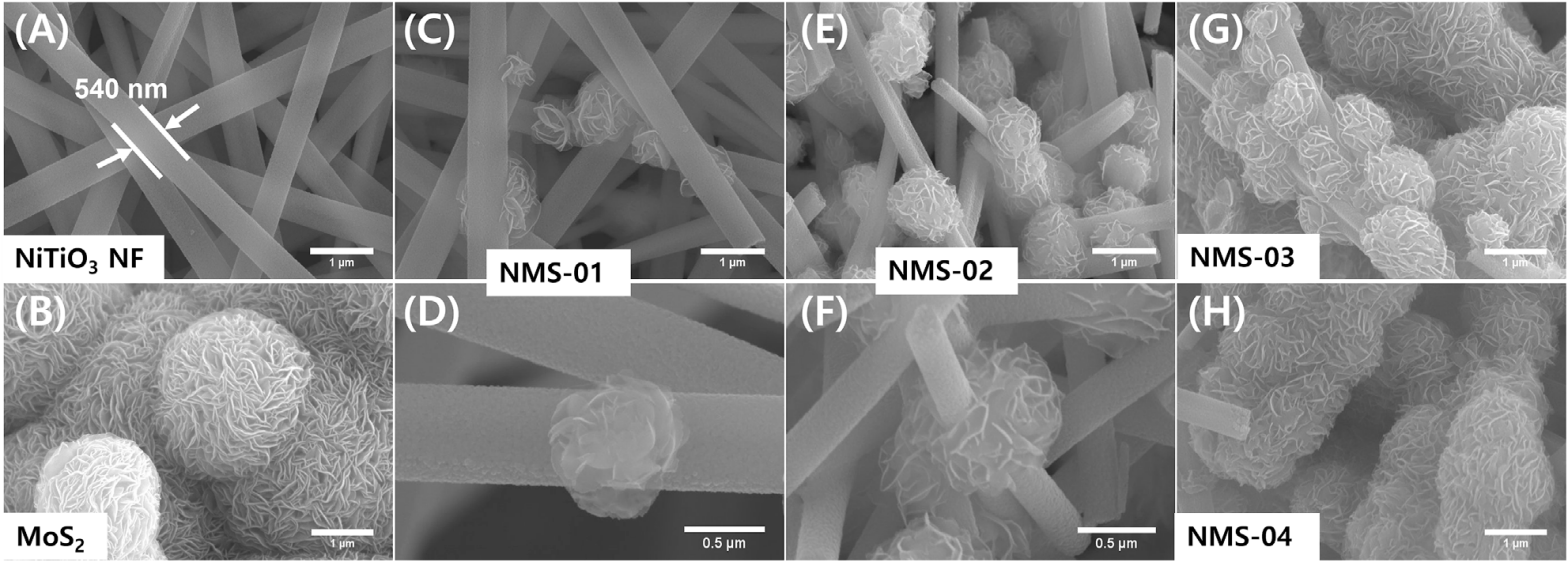
Field-emission electron microscopy images of microstructures. **(A)** Pristine NiTiO_3_ nanofibers, **(B)** pristine MoS_2_, **(C)** low and **(D)** high magnification images of NMS-01, **(E)** low and **(F)** high magnification images of NMS-02, **(G)** agglomerated MoS_2_ in NMS-03, and **(H)** NMS-04.

The morphological properties of the as-prepared samples were analyzed by FE-SEM **(** Figure 1 **)**. The pristine NiTiO_3_ NFs **(** Figure 1A **)** had a smooth surface, with an average diameter of 540 nm and average length of a few micrometers. Pristine MoS_2_ had a petal-like hierarchical architecture **(** Figure 1B **)**. Hybrid structures (NiTiO_3_/MoS_2_) were synthesized via a hydrothermal method, by varying the loading amount of MoS_2_ precursors. Consequently, the hybrid structures appeared as high aspect ratio NiTiO_3_ NFs of uniform diameter, covered by flower-like MoS_2_ particles formed by sulfurization of MoO_3_. Loading amounts of MoS_2_ were varied and the corresponding hybrid structures were assigned the names NMS-01, NMS-02, NMS-03, and NMS-04, respectively. Growth of flower-like MoS_2_ increased with increasing amounts of MoS_2_ precursor. Optimal growth was observed for NMS-02 **(** Figures 1E,F); higher loadings of MoS_2_ in NMS-03 and NMS-04 resulted in agglomeration **(** Figures 1G,H **)**.

### Transmission Electron Microscopy

**FIGURE 2:**
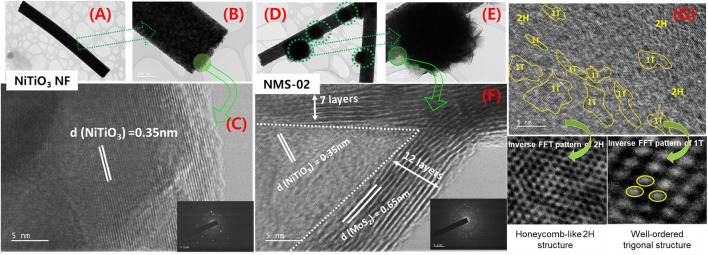
Transmission electron microscope images of microstructures. Pristine NiTiO_3_ nanofibers at **(A,B)** low and **(C)** high resolution indicating interplanar d-spacing, NMS-02 hybrid structure at **(D,E)** low and **(F)** high resolution showing interplanar d-spacings and the number of layers of MoS_2_, and **(G)** inverse fast Fourier transform patterns revealing the co-existence of honeycomb-like 2H and well-ordered trigonal structures in the NMS-02 hybrid structure.

To further investigate the microstructure, TEM analyses were performed on pristine NiTiO_3_ NFs and the optimum hybrid sample (NMS-02). Low-magnification TEM images **(** Figures 2A,B **)** revealed the uniform size and long length of the pristine NFs. Characteristic interplanar spacing of 0.35 nm was confirmed by a high-magnification image of NiTiO_3_ NFs **(** Figure 2C **)**. Low-magnification images of NMS-02 **(** Figures 2D,E **)** show growth of flower-like MoS_2_ firmly attached to the NiTiO_3_ NF substrate. The high-resolution TEM image **(** Figure 2F **)** shows lattice fringes of NiTiO_3_ NFs and MoS_2_ with d-spacings of 0.35 and 0.65 nm, respectively, indicating intimate interfacial contact. Layers (7–12) of MoS_2_ covered the NiTiO_3_ NFs. This analysis confirmed the successful formation of a 1D/2D (NiTiO_3_/MoS_2_) hybrid structure. High-resolution TEM images confirmed the coexistence of honeycomb-like 2H and well-ordered trigonal 1T phases. The 1T/2H hybrid structure was directly observed via selected area inverse fast Fourier transform (FTT) **(** Figure 2G **)**. Insertion of the 1T (metallic) phase into the 2H phase (semiconductor) enhanced the catalytically active site in the composite structure.

### X-ray Diffraction

**FIGURE 3:**
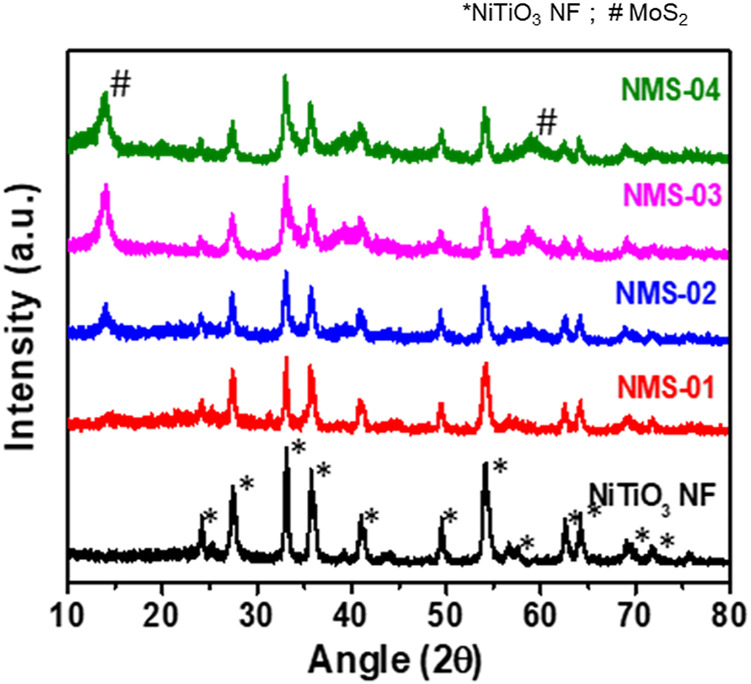
X-ray diffraction patterns of pristine NiTiO_3_ nanofibers and NiTiO_3_/MoS_2_ hybrid structures (NMS-01, NMS-02, NMS-03, and NMS-04).

The phase structures of the samples were characterized by X-ray diffraction (Figure 3 **)**. Pristine NiTiO_3_ showed characteristic peaks at 2θ = 24.09°, 33.06°, 35.73°, 40.96°, 49.40°, 54.08°, 62.45°, 64.03°, and 71° that were assigned to the (012), (104), (110), (113), (024), (116), (124), (300), and (1010) planes of NiTiO_3_ NFs (JCPDS No. 01-076-0334). In addition to the intense peaks of the NiTiO_3_ NFs, a peak due to rutile TiO_2_ appeared at 27.37° (JCPDS No. 98-000-0375). All hybrid samples contained some additional peaks at 2θ = 14.1°, 39.41°, and 58.69°, which were attributed to the (002), (103), and (110) planes of MoS_2_ (JCPDS 01-075-1539). The presence of all characteristic peaks of NiTiO_3_ NFs and MoS_2_ in the XRD spectra of the composite samples confirmed successful integration of the NiTiO_3_/MoS_2_ hybrid structure, in agreement with the TEM results.

### X-ray Photoelectron Spectroscopy

**FIGURE 4:**
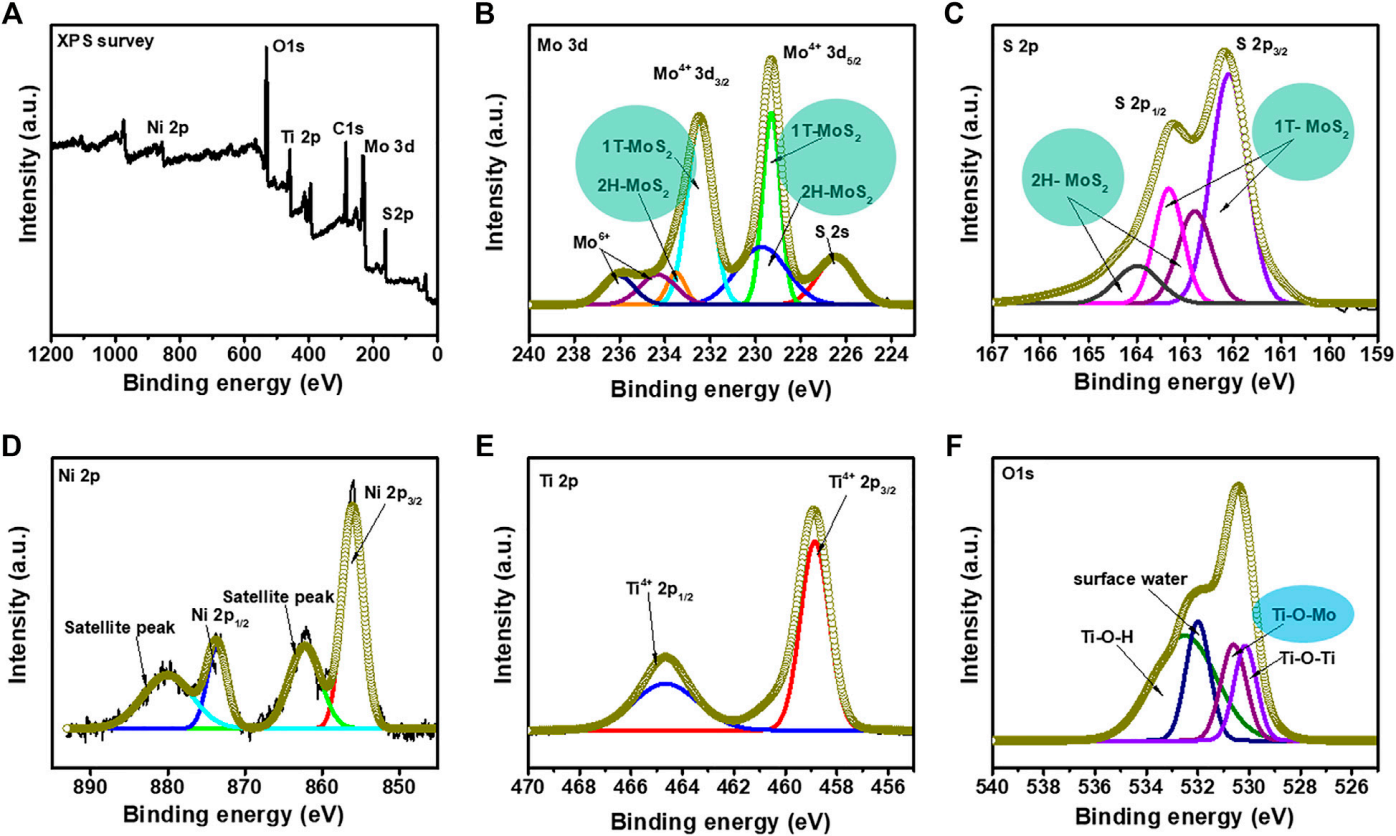
X-ray photoelectron spectra of NMS-2 **(A)** Survey spectrum. Deconvoluted spectra of **(B)** Mo 3d band showing characteristic peaks of the 1T and 2H MoS_2_ phases **(C)** S 2p band showing characteristic peaks of the 1T and 2H phases of MoS_2_, **(D)** Ni 2p band **(E)** Ti 2p band, and **(F)** O 1s band confirming strong interaction between the NiTiO_3_ nanofibers and MoS_2_ constituents in the hybrid structure.

XPS was performed to investigate the chemical composition and effect of MoS_2_ loading on the NiTiO_3_ NFs **(** Figure 4 **)**. Survey spectra **(** Figure 4A **)** confirmed the presence of all essential elements (Ni, Ti, O, Mo, and S) in the NiTiO_3_/MoS_2_ hybrid structure, in correspondence with SEM mapping results (Supplementary Figure S3 **)**. The NMS-02 spectra were further analyzed to investigate the chemical states and interaction between NiTiO_3_ and MoS_2_. The Mo 3d signal **(** Figure 4B **)** consisted of two prominent peaks related to Mo^4+^. High-intensity peaks at 229.3 and 232.4 eV, corresponding to Mo^4+^ 3d_5/2_ and Mo^4+^ 3d_3/2_, respectively, were attributed to the 1T MoS_2_ phase; two other peaks at 230.1 and 233.3 eV were assigned to the 2H phases of MoS_2_. A pair of peaks at 234.2 and 236.0 eV were attributed to Mo^6+^ of MoO_3_. One additional peak at 226.4 eV was assigned to S 2s. The S 2p spectrum **(** Figure 4C **)** was deconvoluted into four peaks having energies of 162.0, 162.7, 163.3, and 164.1 eV. Peaks at 162.0 and 163.3 eV corresponding to S 2p_3/2_ and S 2p_1/2_ were attributed to 1T-MoS_2_ while the peaks at 162.7 and 164.1 eV corresponding to S 2p_3/2_ and S 2p_1/2_, respectively, were attributed to 2H-MoS_2_. The Ni 2p spectrum was deconvoluted into two major peaks corresponding to Ni 3p_3/2_ and Ni 2p_1/2_ at 855.9 and 873.7 eV, respectively **(** Figure 4D **)**. Peaks in the Ti 2p spectra appearing at 458.8 and 464.6 eV were assigned to Ti 2p_2/3_ and Ti 2p_1/2_, respectively **(** Figure 4E **)**. Four O 1s peaks **(** Figure 4F **)** at 530.1, 530.6, 531.9, and 532.5 eV were attributed to Ti–O–Ti, Ti–O–Mo, surface water, and Ti–O–H bonds, respectively. The appearance of the peak at 530.6 eV due to the Ti–O–Mo linkage indicated a strong chemical interaction between NiTiO_3_ and MoS_2_ in NiTiO_3_/MoS_2_, which could improve photocatalytic activity.

### Optical Properties

**FIGURE 5:**
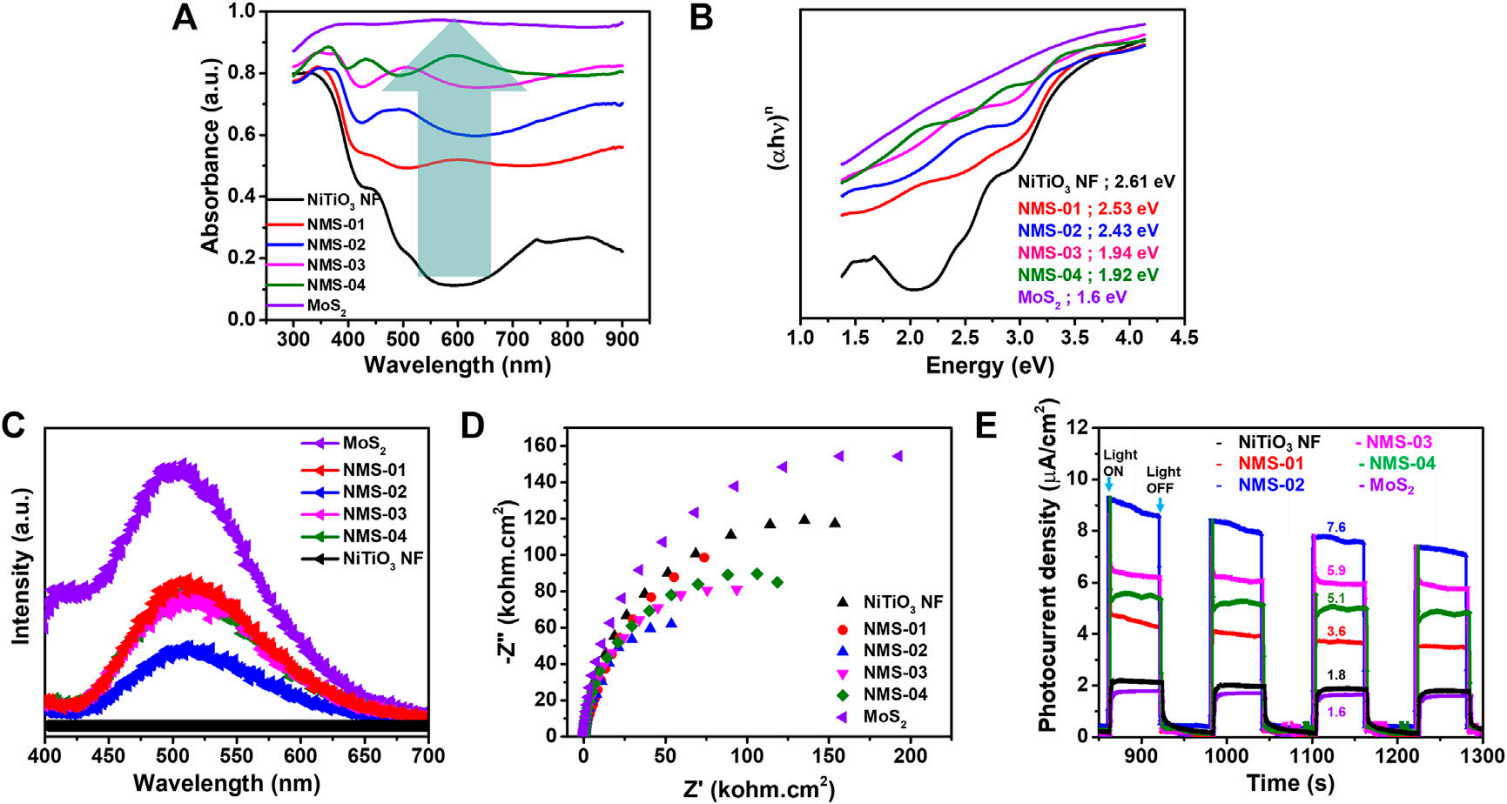
**(A)** Ultraviolet-visible light absorption **(B)** bandgap energy, **(C)** photoluminescence, and **(D)** electrochemical impedance spectra expressed as Nyquist plots, **(E)** photocurrent density measurements for the pristine NiTiO_3_ nanofibers and various hybrid structures.

The optical properties of the as-prepared samples were measured by UV-vis spectroscopy over the range of 300–900 nm **(** Figure 5A **)**. Pristine NiTiO_3_ NFs displayed an absorption edge of 474 nm with little light absorption, especially in the visible region. The light absorption edge red-shifted from 489 nm (NMS-01) to 636 nm (NMS-04) with increasing loading of MoS_2_ onto the NiTiO_3_ NFs. The optimum sample (NMS-02), with an absorption edge of 509 nm, showed more light absorption than pristine NiTiO_3_ NFs, confirming the structural advantage of the composite sample. This enhanced UV-Vis light absorption could promote photocatalytic activity. The bandgap energies estimated using the Tauc method **(** Figure 5B **)** were 2.61, 2.53, 2.43, 1.94, 1.92, and 1.6 eV for NiTiO_3_ NF, NMS-01, NMS-02, NMS-03, NMS-04, and pristine MoS_2_ photocatalysts, respectively. This steady reduction in bandgap energy was attributed to the inherent light absorption of the black MoS_2_.

### Photoluminescence and Photoelectrochemical Performance

For a photocatalyst to show high performance, low rates of recombination of the charge carriers are crucial. Photoluminescence analysis was performed at an excitation wavelength of 325 nm to study the effect of MoS_2_ loading on charge carrier recombination, to design an efficient heterostructure **(** Figure 5C **)**. Pristine NiTiO_3_ NFs showed no intense peaks, which indicated no activation in this region, while MoS_2_ showed high PL intensity due to high recombination rates. A remarkable reduction in charge carrier recombination was found, reflected in quenching of PL peak intensity when MoS_2_ was added to the NiTiO_3_ NFs. The lowest peak intensity for NMS-02 indicated the significance of identifying the optimum amount of MoS_2_, to design an efficient heterostructure to restrain charge carrier recombination. The decreased PL intensity for NMS-02 relative to all of the other photocatalysts was reflected in the highest photocatalytic performance. We performed EIS analysis under UV-vis irradiation to confirm the results obtained by PL spectroscopy, and to study the nature of the charge **(** Figure 5D **)**. A smaller semicircle radius in a Nyquist plot represents lower recombination and more efficient charge transfer across an interface. All of the composite samples showed smaller radii compared with the pristine samples, which confirmed that the growth of MoS_2_ on NiTiO_3_ NFs promoted successful electron transfer, resulting in enhanced photocatalytic performance. The radius was smallest for NMS-02, which also corroborated that it had the best CO_2_ reduction performance. To further confirm its effectiveness of the 1D/2D hybrid structure on the separation of photogenerated electrons and holes, transient photocurrent intensities were measured for bare and hybrid catalysts **(** Figure 5E **)**. The transient photocurrent was measured while switching the light on and off after every 60 s. The photocurrent density of pristine NiTiO_3_ NF and MoS_2_ showed the lowest values of 1.6 μA/cm^2^, and 1.8 μA/cm^2^ respectively while the hybrid structures NMS-01, NMS-02, NMS-03, and NMS-04 showed 3.6 μA/cm^2^, 7.6 μA/cm^2^, 5.9 μA/cm^2^, and 3.6 μA/cm^2^ respectively. This increase in the photocurrent density observed in the hybrid structures, is attributed to the successful formation of heterostructure between 1D NiTiO_3_ NFs and 2D MoS_2_ nanosheets.

### Surface Area and Pore Size Distribution

**FIGURE 6:**
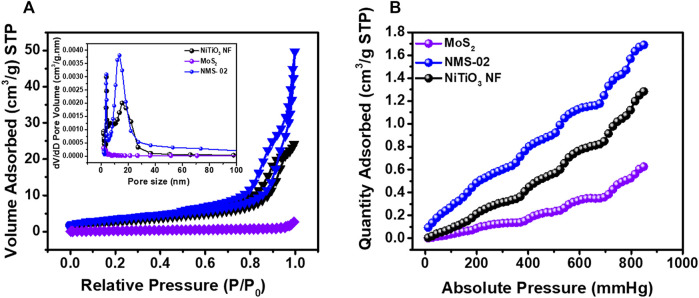
**(A)** Brunauer–Emmett–Teller nitrogen adsorption-desorption isotherms at 77 K. Inset shows the Barrett–Joyner–Halenda pore size distribution. **(B)** CO_2_ adsorption isotherms at 298 K for pristine NiTiO_3_ NF, MoS_2,_ and NMS-02 photocatalysts degassed overnight at 100°C.

Nitrogen adsorption-desorption BET isotherms and Barrett–Joyner–Halenda (BJH) pore size distributions were determined to further investigate the microstructures **(** Figure 6 **)**. All samples were degassed overnight at 100°C prior to analysis. The N_2_ isotherms **(** Figure 6A **)** for MoS_2_ had the smallest specific surface area due to highly densely clustered nanosheets. However, when MoS_2_ sheets were grown on the NiTiO_3_ NFs, the specific surface area significantly increased due to hierarchical “puffy” nanosheets of MoS_2_ dispersed over the surface of the NFs. The higher specific surface area of the composite structure could provide more adsorption and reactive sites, to enhance photocatalytic performance. The BJH pore size distribution plots **(** Figure 6A, inset**)** show typical adsorption-desorption isotherms for NiTiO_3_ and NMS-02 hybrid structures that confirmed the presence of pores, while the isotherms for pristine MoS_2_ indicated the absence of pores. This analysis showed that porous NiTiO_3_ NFs favored the growth of structurally stable vertical nanosheets of MoS_2_. The reduction of CO_2_ in the presence of water is usually in fierce competition with hydrogen evolution reaction (HER). This causes low activity and selectivity toward CO_2_ photoreduction. Therefore, the adsorption and activation of the CO_2_ on the surface of the catalyst are crucial for the subsequent reduction process. The amounts of CO_2_ adsorbed for the TiO_2_ NF, NMS-02, and MoS-02 were analyzed at 25°C **(** Figure 6B **)**. Results show a higher amount of CO_2_ adsorption on the surface of the NMS-02 hybrid sample (1.73 cm^3^/g STP) than that of pristine NiTiO_3_ NF (1.29 cm^3^/g STP) and pristine MoS_2_ (0.62 cm^3^/g STP). The epitaxial growth of MoS_2_ combined with the porous structure of NiTiO_3_ NFs provided more active sites for CO_2_ diffusion and adsorption. FTIR analysis showed the presence of OH groups on the surface of NiTiO_3_ NF and NMS-02 catalysts (Supplementary Figure S4). Surface hydroxyl (OH) and amino groups are prone to donate their protons to CO_2_ to make negatively charged species which help improve CO_2_ adsorption and proton production which enhances the efficiency of CO_2_ photoreduction (Liu P. et al., 2020). The higher adsorption ability of the NiTiO_3_/MoS_2_ hybrid sample supported its high CO_2_ reduction performance. The BET analysis results are summarized in Supplementary Table S2.

### Photocatalytic CO_2_ Photoreduction and its Mechanism

**FIGURE 7:**
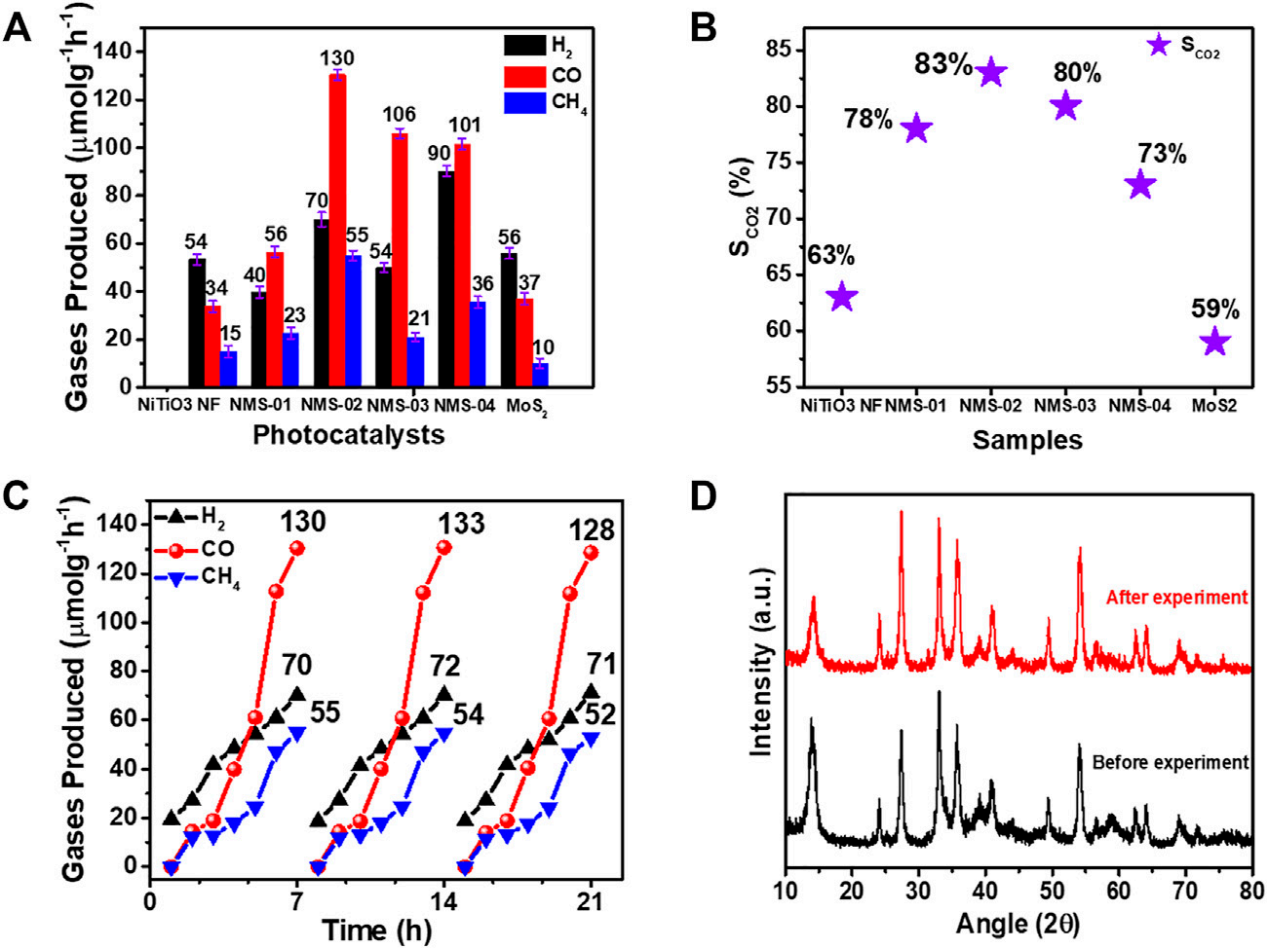
Photocatalytic CO_2_ reduction performance of as-prepared samples. **(A)** Yields of the gases produced. **(B)** Comparison of CO_2_ selectivity among the samples **(C)** Stability test results of the optimum sample (NMS-02).) **(D)** XRD analysis of NMS-02 after the stability test.

To investigate the photocatalytic performance of the samples, CO_2_ photoreduction experiments were performed in a custom-made steel reactor equipped with a quartz window. A 300-W Xe lamp was used as the UV-vis light source. Three sequential experiments were performed under the same conditions to confirm the reliability of the results. Control experiments were also performed without using CO_2_ and photocatalyst; no by-products were obtained, which indicated that photocatalyst and CO_2_ are essential to convert CO_2_ into useful hydrocarbon fuels (Supplementary Figure S1). Figure 7 presents the CO_2_ reduction results. Carbon monoxide was a major gas with comparatively small amounts of H_2_ and CH_4_ as side products. Pristine NiTiO_3_ NF and MoS_2_ showed markedly poorer yields compared with the composite samples, because of their moderate light absorption and charge separation properties. Carbon monoxide and CH_4_ yields increased with increasing MoS_2_ loading and reached the optimum value in NMS-02 (CO: 130 μmol g^−1^ h^−1^; CH_4_: 55 μmol g^−1^ h^−1^). Yields decreased with further increases in MoS_2_ loading to NMS-03 (CO: 106 μmol g^−1^ h^−1^; CH_4_: 21 μmol g^−1^ h^−1^) and NMS-04 (CO: 101 μmol g^−1^ h^−1^; CH_4_: 36 μmol g^−1^ h^−1^), which suggested that excess MoS_2_ might have induced charge carrier recombination during the reaction process. The low activity of the composite samples with higher MoS_2_ contents may also be partly due to fewer active sites, due to agglomerated MoS_2_ sheets as observed in SEM images. The CO and CH_4_ yields of the optimum sample of NMS-02 were 3.8- and 3.6-times those of pristine NiTiO_3_ NF (34 and 15 μmol g^−1^ h^−1^, respectively) and 3.6- and 5.5-times those of the pristine MoS_2_ (37 and 10 μmol g^−1^ h^−1^, respectively) (Figure 7A). The amounts of gases produced by our hybrid samples were significantly higher than those reported elsewhere for photocatalysts containing NiTiO_3_ NFs and MoS_2_ (Supplementary Table S3), because the epitaxial growth of MoS_2_ over NiTiO_3_ NFs enhanced light absorption and exposed active edges. Mixed (1T/2H) phases of MoS_2_, enhanced CO_2_ adsorption, and improved charge separation might also have contributed to the significantly higher performance of the NMS-02 photocatalyst.

$$S_{\mathrm{CO}2\;}\left(\%\right)=\;\frac{2n_{\mathrm{CO}}\;+\;8n_{\mathrm{CH}4}}{2n_{H2}\;+\;2n_{\mathrm{CO}}\;+\;8n_{\mathrm{CH}4}}\;\times 100$$ (1)

Hydrophobic surfaces suppress H_2_ evolution, thereby favoring CO_2_ photoreduction reactions by exerting an umbrella-like effect over photocatalysts to minimize water contact (Li et al., 2019a; Wakerley et al., 2019). CA measurements confirmed that the optimum sample was comparatively hydrophilic compared with pristine NiTiO_3_ NFs and MoS_2_ (Supplementary Figure S2). Here, hydrophobicity could have been partly induced by the presence of the semiconducting hydrophobic 2H phase, and partly by the epitaxial growth of MoS_2_ over the NiTiO_3_ NFs. Generally, 90° is considered a critical angle for distinguishing between hydrophilic (CA < 90°) and hydrophobic (CA ≥ 90°) behavior. However, in our study, surfaces with CAs close to 90° were identified as hydrophobic while those with lower CAs were deemed hydrophilic.

The CO_2_ selectivity (S_CO2_) of each catalyst can be calculated according to Eq. 1, where *n* is the amount of H_2_, CO, and CH_4_ produced in units of μmol g^−1^ h^−1^ during 7 h of light irradiation. The highest selectivity, of 83%, was recorded for NMS-02, which compares with 63%, 78%, 80%, 73%, and 59% for NiTiO_3_ NFs, NMS-01, NMS-03, NMS-04, and MoS_2_, respectively **(** Figure 7B **)**. The stability of the optimum sample was determined by measuring CO_2_ reduction performance. The experiment was repeated for up to three cycles; after each cycle, the sample was removed from the instrument, and heated at 100°C for 4 h to remove DI water and TEOA. No significant change in performance was observed during the three consecutive experiments, which indicated good photocatalyst stability **(** Figure 7C **)**. Moreover, the sample was collected after the completion of the stability test for XRD analysis. Figure 7D clearly shows that the XRD pattern before and after the CO_2_ photoreduction looks almost similar. No observable change was observed according to XRD analysis, confirming that the heterostructure is highly stable.

**FIGURE 8:**
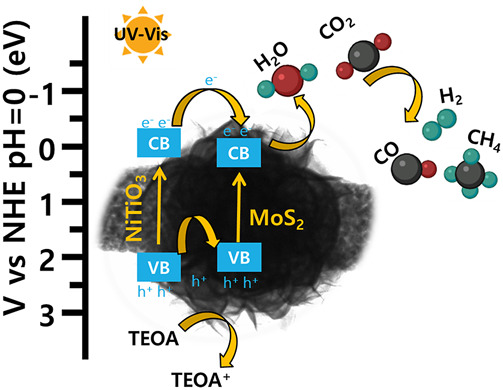
Schematic illustration of the proposed charge transfer mechanism for CO_2_ photoreduction under ultraviolet-visible light illumination on NiTiO_3_/MoS_2_ photocatalyst.

CO_2_ reduction results confirmed that pure NiTiO_3_ NFs and pure MoS_2_ showed exceptionally lower amounts of CO and CH_4_ as compared to those for NiTiO_3_/MoS_2_ heterostructures. It can be inferred that the photocatalytic reduction of CO_2_ can be improved by light-harvesting, photogenerated carrier generating, and the CO_2_ adsorption capacity of the catalyst. Results of UV-Vis absorbance, PL, photocurrent density, and BET analysis show that the introduction of MoS_2_ nanosheets on the surface of NiTiO_3_ nanofibers can effectively increase the light absorption, charge separation, and CO_2_ adsorption and activation ability of the catalyst. As the activated CO_2_ is more susceptible to the reduction, the photogenerated electrons on the surface of heterostructure will react with the activated CO_2_ and H_2_O to form carbon-containing products as well as H_2_. NiTiO_3_ nanofibers decorated with flower-like MoS_2,_ improving the selectivity of the CO, and CH_4_ products through the higher density of the photogenerated electrons to suppress the H_2_ formation on the active sides of the heterostructure. Based on the above results and discussions, a mechanism for the CO_2_ photocatalytic reduction process can be proposed in Figure 8. Under simulated light irradiation, photogenerated electrons are excited from the conduction band (CB) of NiTiO_3_ NFs to the valence band (VB) of NiTiO_3_ nanofibers from where electrons migrate to CB of the MoS_2_ nanosheets due to good band alignment between the two components. Subsequently, the excited electrons from the CB of MoS_2_ nanosheets react with adsorbed CO_2_ and water producing CO and CH_4_. Meanwhile, the holes at VB-holes move from the VB of NiTiO_3_ NF to VB of MoS_2_ where they combine with TEOA to oxidize TEOA to TEOA^+^. The electrons enriched active sites of MoS_2_ in CB would be used for CO_2_ reduction. Thus, the synthesized hybrid structure delayed recombination of electron-hole pairs, thereby improving charge transfer at the interface.

## Conclusion

Full spectrum-induced hybrid structures consisting of 1D NiTiO_3_ decorated with 1T/2H MoS_2_ were prepared via a facile one-step hydrothermal method. The key parameters for tailoring the morphology, porosity, surface, and interfacial properties of the photocatalysts were identified, with a view to efficient and selective conversion of CO_2_ into valuable chemicals. Introduction of MoS_2_ layers onto the NiTiO_3_ NFs increased the CO_2_ selectivity to 83% for the optimized hybrid structure, which compares with 63% and 59% for pristine NiTiO_3_ NFs and MoS_2_, respectively. This large improvement was attributed to the positive synergistic effect between the NiTiO_3_ NFs and MoS_2_ in the hybrid photocatalyst. High CO_2_ selectivity could also be attributed to enhanced light absorption, an abundance of active edges, insertion of multiphase (2H/1T) MoS_2_, and higher surface area, and partly to the hydrophobic nature of the composite structure. We believe that this strategy provides a new route to the design and manufacture of more energy-efficient materials having higher photocatalytic activity.

## Data Availability

The original contributions presented in the study are included in the article/Supplementary material, further inquiries can be directed to the corresponding author.
