# Supplementary material for: Epitaxial Growth of Flower-Like MoS2 on One-Dimensional Nickel Titanate Nanofibers: A “Sweet Spot” for Efficient Photoreduction of Carbon Dioxide
Source: Front Chem. 2022 Jan 27;10:837915. doi: 10.3389/fchem.2022.837915 (PMC8828738; doi:10.3389/fchem.2022.837915)
Supplement: Supplementary file 1 [file DataSheet1.docx]

**Supplementary Information**

Epitaxial Growth of Flower-like MoS_2_ on One-dimensional Nickel Titanate Nanofibers: A “Sweet Spot” for Efficient Photoreduction of Carbon Dioxide

Haritham Khan^1^, Suhee Kang^2^, Hazina Charles^1^, Caroline Sunyong Lee^1, *^

^1^ Department of Materials and Chemical Engineering, Hanyang University, Ansan-si, South Korea

^2^ POSCO Chemical, Sandan-gil, Jeonui-myeon, Sejeong-si, South Korea

***Correspondence:**

**Corresponding Author**

[sunyonglee@hanyang.ac.kr](mailto:sunyonglee@hanyang.ac.kr)


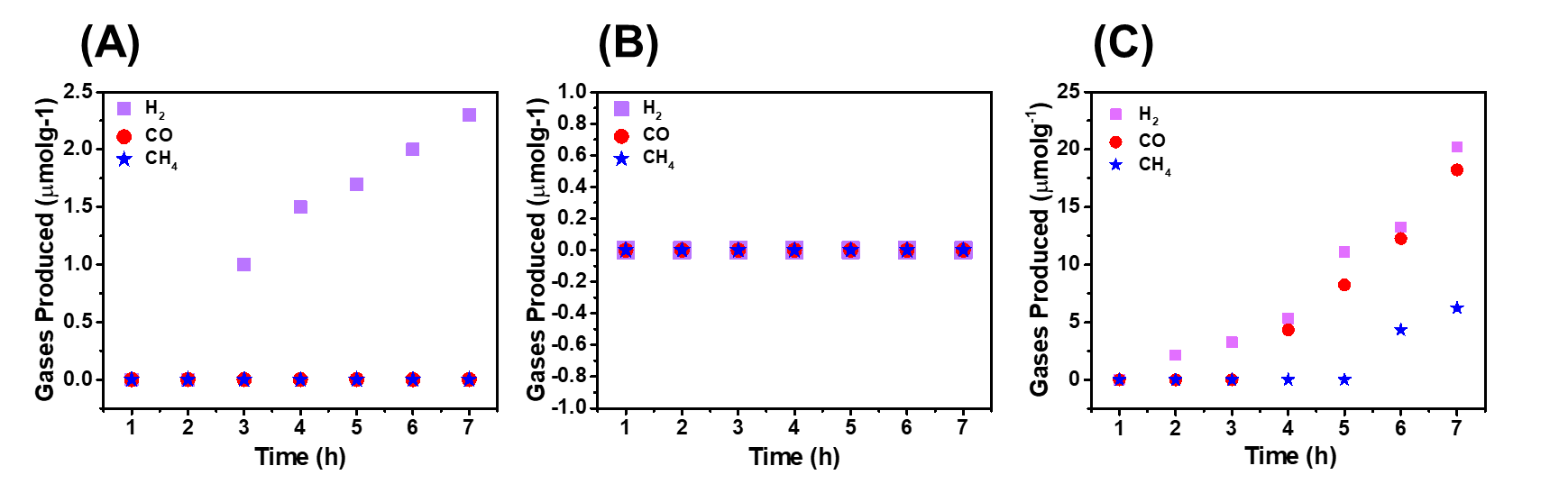


**Figure S1.** Blank tests (A) without CO_2_ gas and (B) without photocatalyst were performed under the same conditions, to verify the origins of the products produced during CO_2_ photoreduction in the presence of NMS-02 photocatalyst. No CO and CH_4_ gases were produced during blank tests in either case, demonstrating that CO_2_ and photocatalysts were prerequisites for the gases to evolve. A small amount of H_2_ gas was produced when the test was performed without CO_2_ gas; this was attributed to water splitting. One another blank test (C) was performed without using TEOA as a sacrificial agent, trace amounts of gases produced during the CO_2_ reduction reveal crucial role of TEOA as a sacrificial agent.


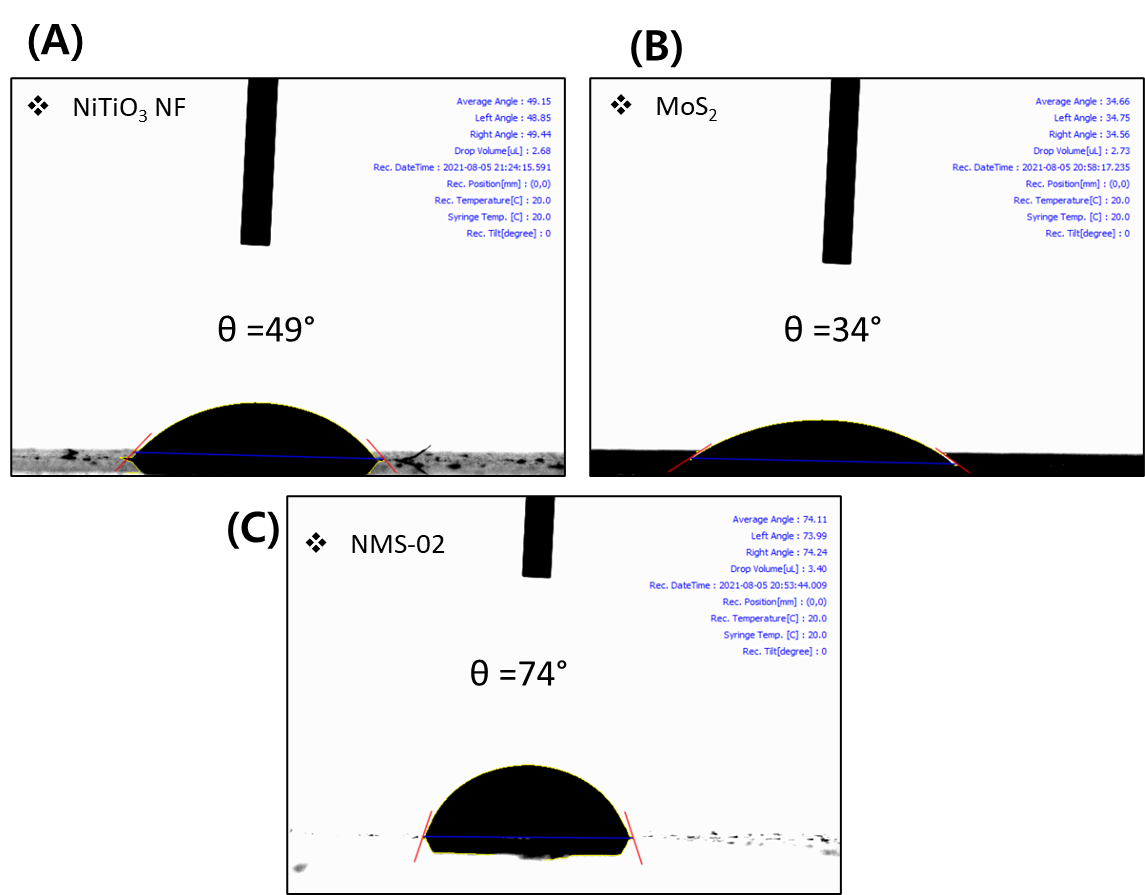


**Figure S2.** Contact angles (CAs) for (A) NiTiO_3_ NFs, (B) MoS_2_, and (C) NMS-02. For the analysis, 3.4 μL of deionized water was used and measurements were repeated five times; the average CA was reported. In our study, CAs close to 90° were considered hydrophobic; lower CAs were considered hydrophilic.


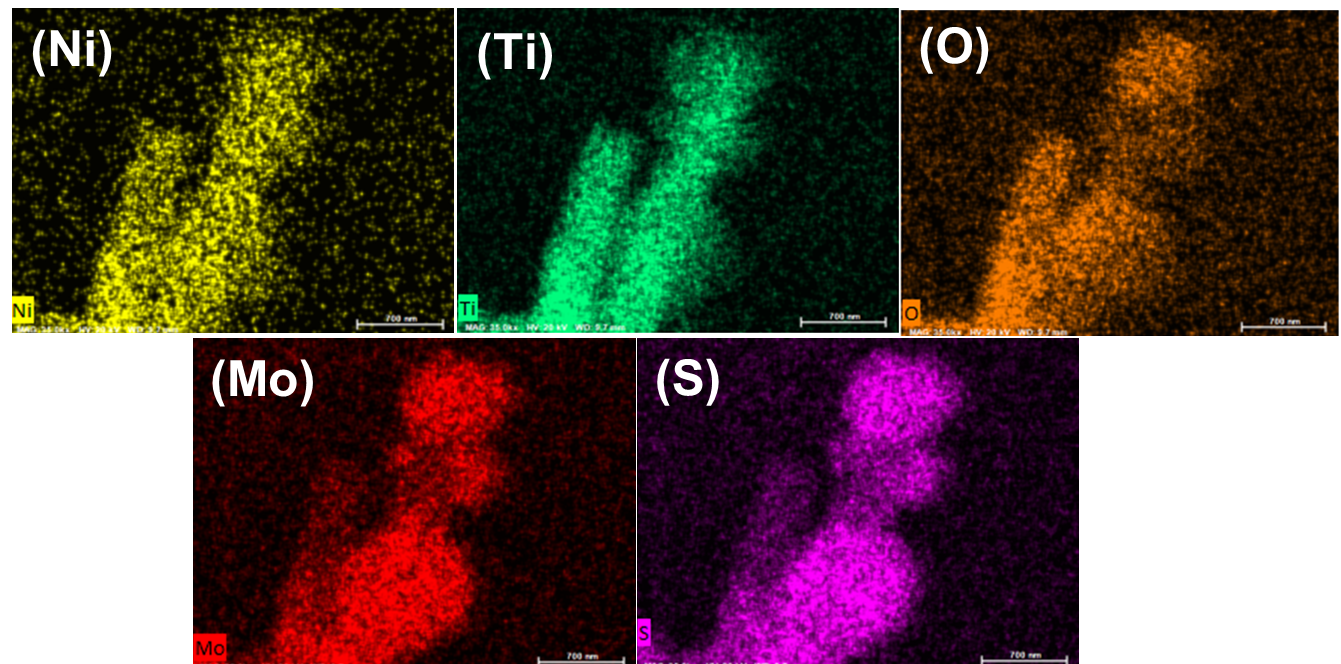


**Figure S3.** SEM (mapping) analysis of NMS-02 photocatalyst. Presence of all essential elements (Ni, Ti, O, Mo, S) on the surface of the hybrid structure indicates successful formation of the heterostructure.


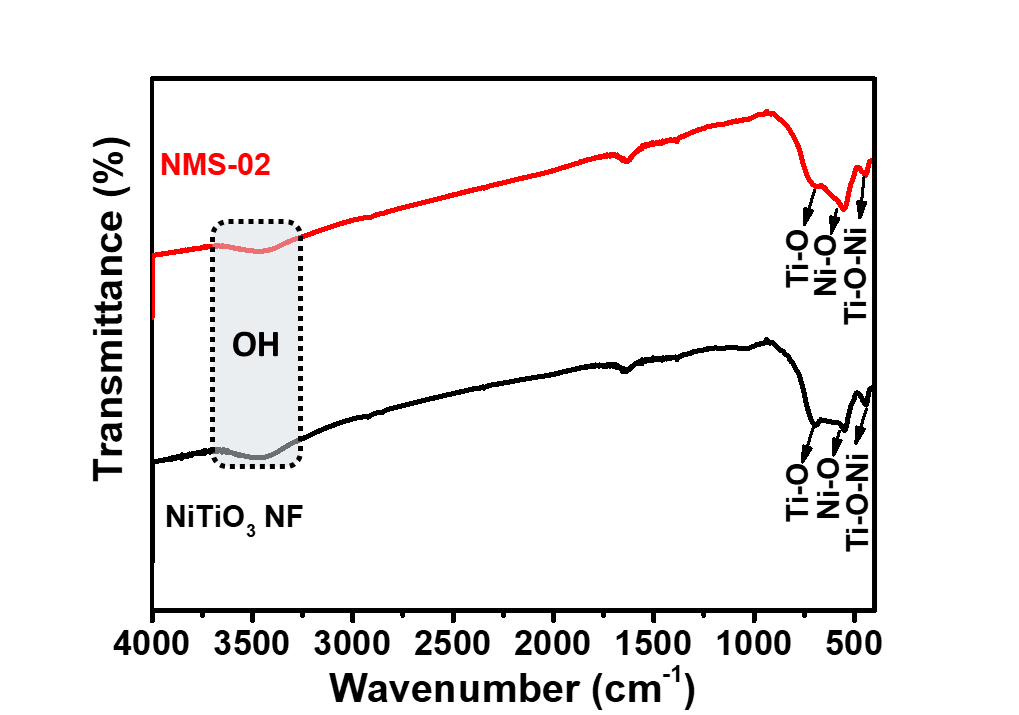


**Figure S4.** FTIR analysis of NiTiO_3_ NF and NMS-02 hybrid structure

**Table S1.** Experimental details of the synthesis of pristine MoS_2_ and the NiTiO_3_/MoS_2_ hybrid samples (NMS-01, NMS-02, NMS-03, and NMS-04) via the hydrothermal process.

| **Na_2_MoO_4_ (g)** | **Thiourea (g)** | **NiTiO_3_ NFs (g)** | **Deionized water (mL)** | **Wt % = [Na_2_MoO_4_ + thiourea]/ [ Na_2_MoO_4_ + thiourea + NiTiO_3_ NF]** | **Sample Name** | |
| --- | --- | --- | --- | --- | --- | --- |
| 0.1 | 0.2 | 0.1 | 60 | 0.75 | NMS-01 |  |
| 0.2 | 0.4 | 0.1 | 60 | 0.85 | NMS-02 |  |
| 0.3 | 0.6 | 0.1 | 60 | 0.90 | NMS-03 |  |
| 0.4 | 0.8 | 0.1 | 60 | 0.92 | NMS-04 |  |
| 0.2 | 0.4 | – | 60 | - | MoS_2_ |  |

**Table S2.** Specific surface area, pore volume, and average pore size of NiTiO_3_ NF, MoS_2_, and NMS-02 structures derived from N_2_ adsorption/desorption and CO_2_ adsorption analyses.

| **Samples** | **S_BET_ (m^2^/g)** | **Pore volume (cm^3^/g)** | **Avg. pore size (nm)** | **CO_2_ adsorption (cm^3^/g)** |
| --- | --- | --- | --- | --- |
| NiTiO_3_ NFs | 13.05 | 0.074 | 23.42 | 1.29 |
| MoS_2_ | 1.06 | 0.006 | 14.34 | 0.62 |
| NiTiO_3_ NFs/MoS_2_ | 18.08 | 0.085 | 17.70 | 1.72 |

**Table S3.** Details of NiTiO_3_ and MoS_2_ photocatalysts based on the literature.

| **S No** | **Photocatalyst** | **Reaction Medium** | **Main products** | **Yield (µmol/g/h)** | **Reference** |
| --- | --- | --- | --- | --- | --- |
| 1 | 10% MoS_2_/TiO_2_ | 300-W Xe lamp | CH_4_, CH_3_OH | 2.86, 2.55 | [1] |
| 2 | TiO_2_/GO/MoS_2_ | 300-W Xe lamp | CO | 92.33 | [2] |
| 3 | Ag/MoS_2_ | 300-W Xe lamp | CO | 75.23 | [3] |
| 4 | SnO_2_/Ag/MoS_2_ | – | CH_4_, CO | 20 µmol, 9 µmol | [4] |
| 5 | CsPbBr_3_/MoS_2_ | 300-W Xe lamp | CH_4_, CO | 41, 116 | [5] |
| 6 | NiTiO_3_/gC_3_N_4_ | 300-W Xe lamp | CH_3_OH | 13.74 | [6] |
| 7 | NiTiO_3_ nanoflowers | 300-W Xe lamp | CH_4_ | 25.8 | [7] |
| 8 | 1D NiTiO_3_ nanofibers/MoS_2_ | 300-W Xe lamp | CO, CH_4_ | 130, 55 | This work |

[1]. Adv. Optical Mater. 2018, 6, 1800911

[2]. ACS Sustainable Chem. Eng. 2018, 6, 5718−5724

[3]. Applied Catalysis B: Environmental 271 (2020) 118931

[4]. Journal of Hazardous Materials 381 (2020) 12097

[5]. Chemical Engineering Journal 416 (2021) 128077

[6]- Chemical Engineering Journal 412 (2021) 128646

[7]- Y. Wang et al. / Energy 169 (2019) 580e586
